# Severe but reversible pulmonary hypertension in scleromyxedema and multiple myeloma: a case report

**DOI:** 10.1186/s12890-019-1020-6

**Published:** 2020-01-09

**Authors:** Mazen Kreidy, Ali Al-Hilli, Ralph Yachoui, Jeffrey Resnick

**Affiliations:** 1 Department of Pulmonary and Critical Care Medicine, Marshfield Clinic, Marshfield, WI USA; 2 Department of Internal Medicine, Marshfield Clinic, Marshfield, WI USA; 3 Department of Rheumatology, Ronald Reagan UCLA Medical Center, Santa Monica, California, USA; 4 Department of Pathology, Marshfield Clinic, Marshfield, WI USA; 5 Present affiliation: Christiana Care Health System, PO Box 1668, Wilmington, DE 19899 USA

**Keywords:** Scleromyxedema, Pulmonary hypertension, Multiple myeloma, Bortezomib, Cyclophosphamide, Dexamethasone

## Abstract

**Background:**

Scleromyxedema is a progressive, systemic connective tissue disorder characterized by fibro-mucous skin lesions and increased serum monoclonal immunoglobulin levels. Pulmonary involvement occurs in a subset of patients, though the overall prevalence of pulmonary lesions in scleromyxedema is unknown. Since pulmonary hypertension presumably occurs in these patients due to disease progression and development of additional conditions, treatment of the underlying plasma cell dyscrasia and connective tissue disorder may improve pulmonary hypertension symptoms.

**Case presentation:**

An elderly patient with scleromyxedema developed pulmonary hypertension refractory to vasodilator and diuretic therapy and subsequently multiple myeloma that responded to a combination therapy of bortezomib, cyclophosphamide, and dexamethasone treatment.

**Conclusions:**

Treatment of the underlying disease(s) that contributed to pulmonary hypertension development with anti-neoplastic agents like bortezomib may improve cardiopulmonary symptoms secondary to reducing abnormal blood cell counts and paraprotein levels.

## Background

Scleromyxedema (papular mucinosis, generalized lichen myxedematous, Arndt-Gordon disease) is a rare, chronic, progressive disorder characterized by skin lesions with mucinous material deposition, fibrosis, increased population of fibroblasts, and high levels of immunoglobulins (monoclonal gammopathy of unknown significance [MGUS]) in serum without a corresponding thyroid abnormality [1–11]. In a subset of cases described by Rongioletti et al., immune cell infiltrates may also be present in skin lesions in a “granuloma annulare-like pattern” [7]. Scleromyxedema is a multi-organ disorder that can involve the nervous system, lungs, heart, kidneys, esophagus, larynx, eyes, muscles, bone marrow, and skin [2–11]. Death can occur due to organ involvement or evolution into a blood malignancy or other cancer [2–4, 6, 8, 10–19]. In a 2013 retrospective study of 30 scleromyxedema cases, two patients died from Hodgkin lymphoma or myeloid leukemia at 22 months and 11 years post-scleromyxedema diagnosis, respectively, without undergoing melphalan treatment (a chemotherapeutic agent associated with development of hematological malignancies) [2, 3, 6, 8, 12]. Treatment generally involves modulating the immune system, decreasing the population of plasma cells, and/or improving dermatological manifestations of the disease; pharmacological and surgical treatments include intravenous immunoglobulin (IVIG), thalidomide/thalidomide derivative lealidomide, systemic glucocorticoids, melphalan, bortezomib plus dexamethasone, and autologous stem cell transplantation among others [2–6, 8–14, 16–41].

As a dermatological condition with disease characteristics similar to scleroderma, scleromyxedema is generally classified as a connective tissue disorder with associated immune system/inflammatory responses, but due to the abnormal amounts of monoclonal immunoglobulin produced (paraproteinemia) and associated abnormal plasma cell populations, scleromyxedema is also considered a paraneoplastic and hematologic disorder [1–11, 36]. In these contexts, many scleromyxedema-associated syndromes can develop, including pulmonary hypertension (PH), myeloproliferative neoplasms (MPN), leukemia/lymphoma, and multiple myeloma [1, 6, 8–22, 24–27, 29, 31–33, 35–37, 39–41]. PH, defined by a mean pulmonary arterial pressure of ≥25 mmHg at rest, can also occur in patients with MPN and other paraneoplastic conditions, and it is demonstrated in the medical literature that PH symptoms improve in response to treatment when the underlying MPN is targeted [25, 42–63]. Therefore, prescribing treatment regimens that target the overlapping pathophysiological characteristics of these associated conditions may simultaneously improve symptomology in patients with multi-system scleromyxedema [2, 13, 17, 19–22, 24–26, 36].

In this case report, we describe the diagnosis, treatment, and progression of PH in a patient who had scleromyxedema and developed multiple myeloma refractory to triple PH treatment that resolved with a combination treatment of bortezomib, cyclophosphamide, and dexamethasone. We propose that combination therapy with the anti-neoplastic agent bortezomib is an important adjuvant therapy to reverse vasodilator resistant PH in patients with scleromyxedema and plasma cell dyscrasias.

### Case presentation

**Fig. 1:**
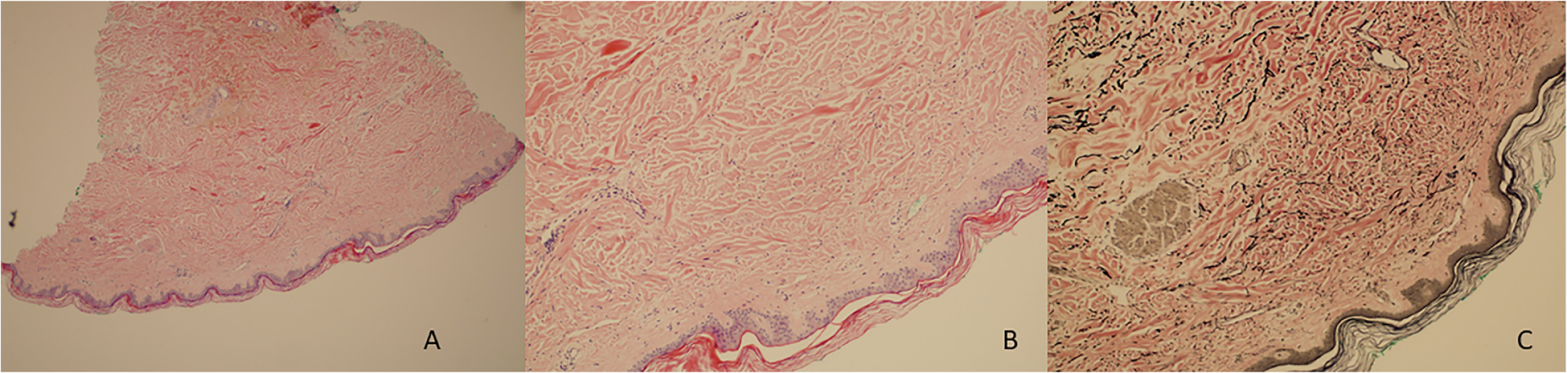
Pathology analysis of skin biopsies highlighting fibrosis and fibrocytic proliferation indicative of scleromyxedema using a Hematoxylin and Eosin stain, b colloidal iron staining for mucin deposition, and c Verhoeff van Gieson elastic stain

A 77-year-old male nonsmoker with a history of atrial fibrillation and sick sinus syndrome post-pacemaker placement experienced edema, skin rash, and skin tightening. During the initial physical examination, yellow-brown papules and indurated and pendulous skin folds were evident on his face, neck, retroauricular area, chest, trunk, upper extremities, and thighs; he also had difficulty opening his mouth. No gross changes were observed in the digital nail beds (i.e., pitting and capillary loops) but were present in the lower extremities. Edema was present in the lower extremities. Skin biopsies revealed fibrosis and benign fibrocytic proliferation consistent with scleromyxedema (Fig. 1a). Colloidal iron staining for mucin deposition detected minimal interstitial mucin deposition in one biopsy, although Verhoeff van Gieson elastic stain highlighted fragmentation of superficial dermal collagen bundles (Fig. 1b and c). Congo red stain for amyloid deposition was negative. Aside from telangiectasia, there was no evidence of vasculopathy or thrombosis associated with these lesions. Laboratory testing of serum and urine samples detected an elevated level of immunoglobulin G (IgG) production, consistent with a diagnosis of MGUS. Electrophoresis of serum proteins revealed a total IgG level of 1500 mg/dL. Elevated levels of two IgG lambda monoclonal antibodies (~ 0.4 g/dL each) with a kappa to lambda ratio of 0.28 were detected by immunofixation of serum samples. The remaining blood values were normal for hemoglobin and calcium levels, but the patient’s kidney function was slightly above normal (creatinine level was 1.4 mg/dL and estimated glomerular filtration rate [eGFR] was 54 mL/min/1.73 m^2^). A 24-h urine sample was positive for lambda Bence-Jones protein at levels too low to quantitate by immunofixation. Although a skeletal survey was negative for lytic lesions, bone marrow biopsies contained 4.7% of mature looking plasma cells.

Additional diagnostic work-up did not suggest multi-organ involvement with scleromyxedema; initial spirometry testing and diffusion lung capacity for carbon monoxide (DLCO) were within normal limits. An echocardiogram indicated the patient had diastolic dysfunction but otherwise normal right and left cardiac function and size with a normal pulmonary artery systolic pressure of 27 mmHg. He was placed on intravenous immunoglobulin G therapy at a dosage of 40 g/mL administered every 6 weeks, with a daily regimen of 60 mg prednisone and 200 mg hydroxychloroquine twice daily. There was significant improvement in the patient’s skin symptoms, and 8 weeks later, his prednisone regimen was weaned down to 5 mg per day. The patient was also started on a thalidomide regimen with an initial dose of 100 mg per day. His IgG levels decreased to 600 mg/dL after one year of maintenance therapy with this regimen.

Over the following 4 years post-scleromyxedema diagnosis, the patient had three recurrences of dermatological symptoms of increasing severity. Episodes of acute symptoms were managed by a burst dose and tapering of steroid medication (prednisone at 60 mg daily until resolution of symptoms then a rapid taper to a maintenance dose of 5 mg daily) and increased dose of IVIG. Thalidomide treatment was discontinued 2 years later due to neuropathy, and hydroxychloroquine treatment was considered inefficient for ameliorating symptoms. During this period of time, the patient’s total IgG levels slowly increased to 1700 mg/dL with concurrent elevations in lambda monoclonal proteins ranging from 0.4–0.5 mg/dL and 0.6–0.7 mg/dL.

At 4 years post-diagnosis, he experienced an acute episode of skin symptoms and severe dyspnea. Severely elevated levels of brain natriuretic peptide (BNP) (2650 pg/mL), indicative of cardiac strain, were detected in serum, and echocardiographic analysis revealed an enlarged right heart with depressed systolic function and an elevated pulmonary arterial systolic pressure estimated at 70 mmHg. Left ventricular function and size was normal. Abnormal pulmonary hemodynamics (in mm Hg) were measured by right heart catheterization, specifically, pulmonary artery pressures of 66/30/42, wedge pressure of 12, right ventricular pressures at 66/15, and right atrial pressure at 13. The pulmonary vascular resistance was estimated at 8.2 international units (IU), while the cardiac output was elevated at 3.65 L/min. Pulmonary function testing revealed a low DLCO at 50%. A chest computed tomography (CT) scan excluded embolism and parenchymal lung disease as contributing factors to elevated right heart dimensions and pulmonary hemodynamics. Additional laboratory testing of serum proteins detected elevated levels of IgG proteins (3670 mg/dL) and the two lambda monoclonal proteins (1.6 g/dL and 1.3 g/dL). A follow-up bone marrow biopsy revealed an ~ 10% normal appearing population of plasma cells that were considered reactive to the patient’s underlying scleromyxedema.

Based on the cardiovascular, pulmonary, and hematological analyses, the patient was diagnosed with a scleromyxedema flare with associated pulmonary arterial hypertension (PAH). He was initially placed on a dual treatment regimen for PAH consisting of 40 mg tadalafil once daily and ambrisentan 5 mg daily that was later increased to 10 mg daily. In addition to increasing the ambrisentan dosage, a daily dose of 40 mg lasix was added to the PAH treatment. Scleromyxedema treatment was optimized with the addition of intravenous chimeric antibodies against CD20 (rituximab) at a dosage and frequency similar to a protocol for rheumatoid arthritis, specifically 1 g of rituximab on days 1 and 15 of the treatment cycle over a period of 24 weeks for a total regimen of three cycles. A burst-taper dose of prednisone was also administered (60 mg, tapered over the next 8 weeks). Over the following year, inhaled trepostinil (vasodilator) was added to the PAH regimen for persistently elevated pulmonary arterial systolic pressure at 42 mmHg and right heart strain on cardiac echography.

On this treatment regimen, the patient’s acute symptoms improved, and he maintained a New York Heart Association (NYHA) functional status of class II. Although the patient’s serum BNP levels decreased to 300 pg/mL, echocardiographic analysis continued to show depressed right heart function and elevated pulmonary arterial pressure at 43 mmHg. A polysomnographic analysis indicated the patient had developed obstructive sleep apnea (Apnea–Hypopnea Index [AHI] of 24 events/hour), and he was subsequently treated with continuous positive airway pressure (CPAP) at 10 cmH2O. Intravenous prostacyclin therapy was considered for PAH, but the patient declined. Follow-up immunoglobulin analysis revealed decreased IgG levels (2060 mg/dL).

Despite maintenance therapy with IVIG and rituximab, the patient developed another severe recurrence of his skin symptoms and worsening dyspnea at 6 years post-scleromyxedema diagnosis (2 years post-PAH diagnosis). Follow-up echocardiography revealed a new left ventricular cardiomyopathy with an ejection fraction of 40%, persistent elevated pulmonary arterial pressure at 44 mmHg, and persistent right ventricular dilation. Serum BNP levels were elevated at 631 pg/mL, and IgG levels had increased to 3420 mg/dL with concurrent elevations of the two monoclonal lambda proteins at 1.48 and 0.37 g/dL. A follow-up bone marrow biopsy revealed an abnormal plasma cell population of 60% consistent with a hematological abnormality. The patient was diagnosed with multiple myeloma associated with an acute episode of scleromyxedema flare up with multi-organ involvement. A treatment regimen of bortezomib (2 mg; dose adjusted per cycle depending on patient-related factors as denoted in Table 1) and dexamethasone (20 mg) (4 weeks per cycle of therapy) was initiated to decrease the plasma cell population, and IVIG treatment was continued to alleviate dermatological symptoms. Over the following 2 years, the patient received a total of seven cycles of bortezomib and dexamethasone (Table 1). There was a dramatic improvement in his PAH, cardiovascular, and dermatological symptoms. Serum analysis revealed decreased BNP and IgG levels at 100 pg/mL and 1300 mg, respectively. A repeat echocardiogram revealed significant improvement in right ventricular size and function as well as left ventricular function, but pulmonary arterial systolic pressure was still elevated at 51 mmHg. However, bortezomib had to be discontinued after the seventh cycle due to worsening neuropathy. A treatment regimen with a lenalidomide derivative (Revlimid) was attempted but also discontinued after 2 months of treatment due to adverse side effects. The patient elected to halt the inhaled trepostinil regimen.

**Table 1:** Chemotherapeutic regimens administered with bortezomib for treatment of multiple myeloma and pre-existing scleromyxedema with pulmonary symptoms

Cycle #	Agent 1	Dose	Agent 2	Dose	Agent 3	Dose	Agent 4	Dose
1	Bortezomib D1, D8, D15	2.5 IV	Dexamethasone Weekly	20 mg PO	IVIG Weekly	40 g IV	–	–
2	Bortezomib D1, D8, D15	2.6 S/Q	Dexamethasone Weekly	20 mg PO	IVIG Weekly	40 g IV	–	–
3	Bortezomib D1, D8, D15	2.6 S/Q	Dexamethasone Weekly	20 mg PO	IVIG Weekly	40 g IV	–	–
4	Bortezomib D1, D8, D15	2.6 S/Q	Dexamethasone Weekly	20 mg PO	IVIG Weekly	40 g IV	–	–
5	Bortezomib D1, D8, D15	2.6 S/Q	Dexamethasone Weekly	20 mg PO	IVIG Weekly	40 g IV	–	–
6	Bortezomib D1, D8, D15	2.6 S/Q	Dexamethasone Weekly	20 mg PO	IVIG Weekly	40 g IV	–	–
7	Bortezomib D1 only, developed neuropathy	2.6 S/Q	Dexamethasone Weekly	20 mg PO	IVIG Weekly	40 g IV		
1	Bortezomib D1, D8, D15	1.5 mg IV	Dexamethasone Weekly	20 mg PO	Cyclophosphamide D1, D8, D15	300 mg IV	IVIG Weekly	40 g IV
2	Bortezomib D1, D8, D15	1.5 mg IV	Dexamethasone Weekly	20 mg PO	Cyclophosphamide D1, D8, D15	300 mg IV	IVIG Weekly	40 g IV
3	Bortezomib D1, D8, D15	1.6 mg IV	Dexamethasone Weekly	20 mg PO	Cyclophosphamide D1, D8, D15	600 mg IV	IVIG Weekly	40 g IV
4	Bortezomib D1, D8, D15	1.6 mg IV	Dexamethasone Weekly	20 mg PO	Cyclophosphamide D1, D8, D15	600 mg IV	IVIG Weekly	40 g IV
Chemotherapy break–		–	Dexamethasone Weekly	20 mg PO	–	–	IVIG Weekly	40 g IV
5	Bortezomib D1, D8, D15	1.2 mg IV	Dexamethasone Weekly	20 mg PO	Cyclophosphamide D1, D8, D15	600 mg IV	IVIG Weekly	40 g IV
6	Bortezomib D1, D8, D15	1.5 mg IV	Dexamethasone Weekly	20 mg PO	Cyclophosphamide D1, D8, D15	480 mg IV	IVIG Weekly	40 g IV
7	Bortezomib D1, D8, D15	2 mg IV	Dexamethasone Weekly	20 mg PO	Cyclophosphamide D1, D8, D15	600 mg IV	IVIG Weekly	40 g IV
8	Bortezomib D1, D8, D15	1.6 mg IV	Dexamethasone Weekly	20 mg PO	Cyclophosphamide D1, D8, D15	600 mg IV	IVIG Weekly	40 g IV
9	Bortezomib D1, D8, D15	2 mg IV	Dexamethasone Weekly	20 mg PO	Cyclophosphamide D1, D8, D15	500 mg IV	IVIG Weekly	40 g IV
	Iron infusion	510 mg IV	–	–	–	–	–	–
	Passed away	N/A	N/A	N/A	N/A	N/A	N/A	N/A

Abbreviations: *D#* Day of treatment cycle, *IV* Intravenous Injection, *IVIG* Intravenous Immunoglobulin, *N/A* Not applicable, *PO* Per Os (oral), *S/Q* Subcutaneos Injection, − unknown

The patient was observed for ~ 14 months before he experienced a recurrence of symptoms and cardiopulmonary decline. His IgG levels had again increased to 2000 mg/dL. The patient was placed on a weekly regimen of 3 mg bortezomib, 20 mg dexamethasone, and 600 mg of cyclophosphamide (Cytoxan) (4 weeks per cycle, last dose omitted because of pancytopenia), and IVIG maintenance therapy was continued at a dosage of 40 g/mL (see Table 1 for dose adjustments per cycle). After four cycles, the patient’s symptoms improved, and his IgG levels decreased to the lowest concentration of 1100 mg/dL. Only one monoclonal lambda protein was detected at 0.52 mg/dL. An echocardiogram revealed normalization of left and right ventricular size and function as well as normalization of pulmonary arterial systolic pressure at 23 mmHg.

After a treatment break of 6 months, the patient’s symptoms recurred, and his IgG levels increased above 2000 mg/dL. The patient underwent five additional cycles of bortezomib, dexamethasone, and cyclophosphamide. His IgG levels stabilized between 2000 and 2500 mg/dL, and a repeat bone marrow biopsy revealed a decrease in the abnormal plasma cell population to 22%. A follow-up echocardiogram revealed normal right and left ventricular size and function and a mildly elevated pulmonary arterial systolic pressure at 38 mmHg. Future plans for the patient’s care involved slowly weaning him from his vasodilator medications; however, he suffered a sudden and fatal out-of-hospital cardiac arrest of unclear etiology at 9 years post-scleromyxedema diagnosis. No autopsy was performed.

## Discussion

Pulmonary hypertension has occurred in association with various hematologic malignancies, particularly those with underlying plasma cell dyscrasias [25, 42–63]. The first case of reversible PH in response to antineoplastic treatment for a scleromyxedema-like condition and hematological malignancy was described by Yaqub et al. in 2004, and in 2015, Feyereisn described the diagnosis, treatment, and outcome of four cases of reversible PH in the setting of plasma cell dyscrasias one of which had scleromyxedema [24, 25]. The overall frequency and spectrum of PH in this setting remains largely undefined.

In our patient with scleromyxedema, multiple anti-neoplastic and immunomodulatory treatment regimens were used to alleviate dermatological and cardiopulmonary symptoms. Immunomodulatory treatments like IVIG, glucocorticoids, and hydroxychloroquine were administered over the entire course of the disease but were unable to produce a complete remission of skin and cardiopulmonary symptoms. Administration of anti-neoplastic agents like thalidomide and bortezomib led to decreased paraprotein levels on multiple occasions and corresponded to improved pulmonary dynamics in a manner similar to previously published cases [24, 25, 27, 60]. Close monitoring and treatment alteration was necessary to prevent unanticipated clinical events. Thalidomide or thalidomide derivatives were used at two points over the course of this patient’s history but were halted due to development of neuropathy and other adverse side effects. Although anti-neoplastic/chemotherapeutic agents can be associated with the development of PH, pulmonary injury, and hematological malignancies, we do not believe this occurred based on the temporal progression of scleromyxedema from a localized cutaneous condition to a generalized disease with multiple phenotypes over a period of 9 years [2–4, 6, 8, 10–12, 47, 53, 64–80]. Furthermore, PH developed 2 years after thalidomide treatment was stopped, and cardiopulmonary symptoms for the most part resolved in response to multiple myeloma treatment. Despite a partial therapeutic response with respect to abnormal plasma cell populations and IgG production, this patient experienced excellent recovery of cardiopulmonary function when on anti-neoplastic treatment regimens. Thus, a complete remission of scleromyxedema and associated plasma cell dyscrasia and paraprotein levels does not appear to be necessary to obtain a significant improvement in PH symptoms.

Although the physiopathology of PH development in response to plasma cell dyscrasias has not been fully elucidated, the reversibility of hemodynamics in response to treatment with chemotherapeutic and immunomodulatory agents offers hope for PAH patients [24, 25, 27, 42, 43, 45, 50, 53, 57–60, 62, 63, 81–103]. Improvements in hematopoietic cell populations, paraprotein levels, and hemodynamic functions in our patient and other cases of reversible PH suggest that abnormal plasma cell populations play a central role in the development of PH [24, 25, 27, 43, 45, 50, 53, 57–60, 62, 63, 81, 84–86, 91]. Furthermore, patients with scleromyxedema and related conditions who received treatments traditionally used for multiple myeloma have exhibited decreases in IgG and paraprotein levels that co-occurred with clinical improvements, as in our patient [17, 20–25, 31, 35, 60, 61]. This may also indicate a direct link between decreased paraprotein levels and hemodynamic improvements by way of improved hyperviscosity and associated microvascular dysfunction [8]. However, detectable paraprotein levels are not always present nor correspond to the severity, progression, and response of scleromyxedema to standard treatments for this condition; therefore, the relative contribution of scleromyxedema progression and paraprotein levels to PH development is unclear [3, 5, 6, 8, 9, 13, 14, 16–18, 20–25, 31, 35, 60, 61]. Other pathobiological mechanisms invoked in the development of scleromyxedema and/or associated with plasma cell dyscrasias may also contribute to the development of PAH. These include increased secretion and expression of cytokines, dysregulation of immune system activities, and/or abnormal pulmonary fibroblasts, mucin deposition in the pulmonary vasculature, and direct invasion of the pulmonary vasculature by abnormal plasma cells [2–4, 8, 11, 14–16, 18, 27, 29, 31–38, 41, 57, 62, 63, 81, 86, 87, 96, 104–111]. Alternatively, a direct effect of chemotherapeutic agents on the pulmonary vasculature and associated abnormal humoral milieus may also have played a role in this reversibility. Due to the multifaceted nature of PH development and reversibility in response to treatment, it is also possible that a multi-hit model, as in idiopathic pulmonary arterial hypertension (IPAH), is plausible [106]. Regardless of the underlying etiology, patients with concurrent PH and MPN or vice versa have demonstrated hemodynamic improvements in response to chemotherapeutic agents; thus, patients with vasodilator-resistant PH may derive benefit from cancer treatments [25, 27, 42–63].

Evidence for a cancer-like pathology and direct effects of immunosuppressive and anti-proliferative agents on PH development and progression is present in case reports for patients with PAH [24, 25, 42, 43, 45–55, 57, 58, 60–63, 76, 81, 85–87, 90–96, 105–107, 110–113]. According to Price et al., “Pathologic specimens from patients with PAH demonstrate an accumulation of perivascular inflammatory cells,” and laboratory analysis of serum from patients with PAH revealed increased levels of cytokines, chemokines as well as autoantibodies to endothelial cells and fibroblasts [105, 108, 109]. In parallel to those abnormalities, the pulmonary vascular cells of patients with PAH exhibit many features of cancerous cells from dysregulated metabolism to increased cell proliferation and resistance to apoptosis [106, 109]. These observations combined with the occurrence of PAH in various connective tissue diseases support the role of inflammation, autoimmunity, and a neoplastic-like dysregulation at the center of the pathogenesis of PAH [105–107, 110–113]. This model could provide a mechanistic explanation for the hemodynamic improvements noted in our patient and in many patients with PAH in response to immunosuppressive and anti-proliferative agents [24, 25, 27, 42–63]. The following paragraphs provide a description of currently used agents for multiple myeloma and/or scleromyxedema that have proven effective for patients with PAH.

Glucocorticoids have an inhibitory effect on multiple types of immune cells and produce broad anti-inflammatory and immunosuppressive effects [114]. Glucocorticoids have been used as a treatment for scleromyxedema with positive effects reported in single case reports, and its efficacy against collagen disease-associated PAH is well known [31–35, 84, 88–90, 112–114]. Glucocorticoids are also used as a first line therapy for multiple myeloma in combination with other chemotherapy regimens in patients ineligible for autologous stem cell transplantation [115]. Improvements post-prednisolone treatment have been noted in adult and pediatric patients with iPAH as well as in monocrotaline-induced pulmonary arterial hypertension in rodents [88–90, 96, 97].

Rituximab is an FDA-approved chimeric anti-CD20 monoclonal antibody for various malignancies and autoimmune disorders [116]. It exerts its immunosuppressive and anti-proliferative effects through antibody- and complement-mediated dependent cellular toxicity and apoptosis and has been used anecdotally for mixed connective tissue disorders [91, 112, 116]. Previously published case reports demonstrated improvements in collagen vascular disease-associated PAH in response to rituximab treatment, and a large randomized placebo controlled clinical trial of rituximab for the treatment of scleroderma-associated PAH is currently underway (ClinicalTrials.gov identifier NCT01086540) [92, 93]. In a presumed case of iPAH, rituximab co-treatment with chemotherapy for lymphoma lead to symptom resolution, and PAH in the setting of Castleman lymphoma was also noted to respond to rituximab [63, 85]. However, in two instances, rituximab use was associated with the development of PH [79, 80].

Plasma exchange or plasmapheresis is an automated technique that permits the selective therapeutic exchange of patient plasma with another fluid. Plasma exchange has been suggested as a treatment for scleromyxedema and was noted to improve PAH in association with various connective tissue diseases [37, 38, 86]. Immunoadsorption (IA), another extracorporeal automated technique to selectively remove immunoglobulins from the plasma of PAH patients via high affinity absorbers, is a promising treatment for iPAH; in 5 patients with severe iPAH awaiting transplant, IA improved symptoms associated with iPAH [94]. IA as an add-on to targeted medical therapy also led to improvements in mean PVR and CI in 10 patients with iPAH though these hemodynamic improvements did not correlate to substantial improvements in the 6 mn walk test [95].

Bortezomib is a proteasome inhibitor that is FDA-approved for the treatment of multiple myeloma [117, 118]. In the medical literature, patients with multiple myeloma and PH can experience reversal of PH symptoms with bortezomib treatment, although adverse pulmonary effects have also been reported in association with this drug [25, 64–71, 73, 75, 76]. Steroid co-treatments can prevent bortezomib-induced lung injury, though additional studies are needed to assess both the adverse pulmonary side effects as well as the protective effect of an adjuvant steroid regimen for bortezomib treatment [64–71, 73, 75, 76]. In animal models of pulmonary disease, bortezomib treatment reverses adverse cardiopulmonary effects and can improve survival post-monocrotaline-induced PH/PAH [98–103]. In a mouse model of hypoxia-induced PH, bortezomib treatment prevented an increase in right ventricular systolic pressure, ratio of right ventricular weight to left ventricular weight and septum (right ventricular hypertrophy index), percent medial wall thickness, and muscularization of pulmonary vessels and inhibited vascular smooth muscle proliferation [98]. A similar treatment effect was observed in rats with monocrotaline- or left-to-right shunt-induced PAH, and bortezomib treatment also enhanced survival in monocrotaline-injected rats compared to monocrotaline-injected rats without bortezomib treatment [98–102]. In a chronic hypoxia-induced PAH rat model, Ibrahim and colleagues noted that anti-tumor agents, specifically bortezomib, MG-132, and daunorubicin, decreased pulmonary vessel thickness and, in the case of daunorubicin and MG-132, improved pulmonary response to vasodilator treatment [103]. Together, these results suggest that proteasome inhibitors alone or in combination with vasodilators could potentially prevent and/or reverse PAH-induced pulmonary vessel remodeling and hemodynamic response in PAH afflicted patients [98–103].

Regardless of the agent selected, it is clear from our and previous cases that PH/PAH can be improved by the addition of anti-neoplastic agents to the overall treatment regimen of patients with conditions that produce plasma cell dyscrasias, abnormal protein levels, and increased extracellular matrix deposition [24, 25, 27, 43, 45, 50, 53, 57–60, 62, 63, 81–103]. However, these therapies can produce adverse side effects that may potentially limit the number and type of treatments available for PH, multiple myeloma, and other conditions associated with multi-system scleromyxedema [2–4, 6, 8, 10–12, 47, 53, 64–80]. Careful monitoring is necessary to mitigate adverse treatment effects in this patient population.

## Conclusion

Although bortezomib and cyclophosphamide are generally used as second- and third-line treatments for scleromyxedema and related cutaneous mucinoses, these agents may be an effective primary therapy for these conditions in combination with glucocorticoids and/or proteasome inhibitors [4, 6, 8, 10, 13, 14, 16–18, 22–24, 26, 27, 30, 69, 87] especially in the presence of plasma cell dyscrasia-associated PAH. Treatment of our patient’s underlying plasma cell abnormality with a combination treatment of cyclophosphamide, bortezomib, and dexamethasone not only reduced the population of abnormal plasma cells in the bone marrow but also improved the dermatological, cardiopulmonary, and paraprotein effects of scleromyxedema and multiple myeloma-induced PH as well similar to a few other previously reported cases [24, 25, 27, 60]. Therefore, a combination regimen of cyclophosphamide, dexamethasone, and bortezomib may be an effective multi-target treatment for patients with PH refractory to vasodilator treatment, in the setting of plasma cell dyscrasias and elevated paraprotein levels. Additional work is necessary to understand the physiology of chemotherapeutic agents for PAH-associated plasma cell dyscrasias and develop treatment regimens to maximize clinical response with minimal side effects.

## Data Availability

Data sharing is not applicable to this article, as no datasets were generated or analyzed during the current study. A complete listing of data from the patient’s electronic medical record is unavailable for viewing, sharing, or dissemination per HIPAA guidelines.

## Abbreviations

AHI: Apnea–Hypopnea Index
BNP: Brain natriuretic peptide
CPAP: Continuous positive airway pressure
CT: Computed tomography
DLCO: Diffusion lung capacity for carbon monoxide
eGFR: Estimated glomerular filtration rate
IgG: Immunoglobulin G
IU: International units
IVIG: Intravenous immunoglobulin
MGUS: Monoclonal gammopathy of unknown significance
MPN: Myeloproliferative neoplasms
NYHA: New York Heart Association
PAH: Pulmonary Arterial Hypertension
PH: Pulmonary Hypertension
POEMS: Polyneuropathy, organomegaly, endocrinopathy/edema, M-protein, skin changes
